# Vaccines against SARS‐CoV‐2 are safe to administer in patients with antibodies to pegaspargase

**DOI:** 10.1002/cam4.5011

**Published:** 2022-07-15

**Authors:** Hope D. Swanson, Hana Hakim, Diego R. Hijano, Ted Morton, Shane Cross, Hiroto Inaba, Sima Jeha, Ching‐Hon Pui, Seth E. Karol

**Affiliations:** ^1^ Department of Pharmacy and Pharmaceutical Sciences St. Jude Children's Research Hospital Memphis Tennessee USA; ^2^ Department of Infectious Diseases St. Jude Children's Research Hospital Memphis Tennessee USA; ^3^ Department of Oncology St. Jude Children's Research Hospital Memphis Tennessee USA; ^4^ Department of Global Pediatric Medicine St. Jude Children's Research Hospital Memphis Tennessee USA

## Abstract

**Objective:**

Allergic reactions to pegaspargase during ALL therapy are typically due to antibodies against polyethylene glycol (PEG), which is also used as a stabilizing agent in mRNA‐based SARS‐CoV‐2 vaccines. To evaluate the safety of these vaccines in patients with anti‐pegaspargase antibodies.

**Methods:**

We retrospectively reviewed the records of patients treated for ALL who had received SARS‐CoV‐2 vaccinations. All patients had antibodies against pegaspargase assayed during ALL therapy prospectively and in response to clinical allergies. Symptoms of intolerance to vaccination were gathered retrospectively from chart abstraction.

**Results:**

SARS‐CoV‐2 vaccination was well tolerated in all 78 patients with prior exposure to pegaspargase as part of their leukemia therapy. No reactions were observed in the 54 patients without a history of anti‐pegaspargase antibodies or in 19 patients with antibodies who received mRNA vaccination. 1 patient who received the polysorbate containing Janssen vaccine experienced mild symptoms after vaccination not meeting the criteria of clinical allergy which spontaneously resolved within 25 minutes.

**Conclusion:**

SARS‐CoV‐2 vaccination is safe in this population.

## Graphical abstract

SARS‐CoV‐2 vaccination is well tolerated in patients with a history of clinical allergy to pegaspargase. This is true for both patients with and without a history of anti‐pegaspargase antibodies.

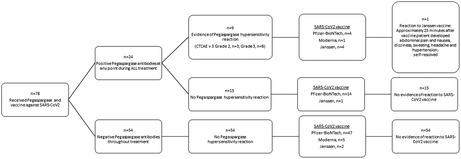

Both Pfizer‐BioNTech and Moderna mRNA COVID‐19 vaccines contain polyethylene glycol (PEG) as an inactive ingredient. Recommendations from the Centers for Disease Control and Prevention list severe allergic reaction to a component of the vaccine as a contraindication to administration.^1^ PEG is thought to be one of the causes of allergic reactions to the mRNA vaccines.^2^, ^3^ Patients treated for acute lymphoblastic leukemia/ lymphoma (ALL) are exposed to PEG in the form of pegaspargase, a critical component of all pediatric ALL therapy. The most common side effect of pegaspargase therapy is a hypersensitivity reaction.^4^, ^5^, ^6^ Such apparent hypersensitivity reactions can reflect either infusion reactions without an identifiable immunologic component or be driven by antibodies to pegaspargase.^4^, ^7^ Because allergy to PEG is the primary driver of allergic reactions to pegaspargase,^7^ there are safety concerns for administering PEG‐containing COVID‐19 vaccines to patients with a history of pegaspargase hypersensitivity reactions during ALL therapy.^8^ However, alternative vaccine options are limited as the Pfizer‐BioNTech is the only SARS‐CoV‐2 vaccine currently available to children. This limitation leaves a substantial number of patients at high risk of severe COVID‐19 disease^1^, ^9^, ^10^, ^11^ without the protection of highly effective vaccines.

Prior publications have reported the safe administration of mRNA vaccines in patients with prior history of hypersensitivity reactions to pegaspargase.^8^, ^12^, ^13^, ^14^ However, the rate of reactions to these vaccines in patients with anti‐PEG antibodies following pegaspargase therapy has not been reported. Prior reports suggest these antibodies develop in more than 80% of patients with clinical hypersensitivity reactions to pegaspargase and 18% of patients receiving pegaspargase without a history of clinical reaction.^7^ We therefore sought to assess the risk of allergic reaction to COVID‐19 vaccination in patients previously exposed to pegaspargase with and without a history of anti‐pegaspargase antibodies.

Patients treated at St. Jude Children's Research Hospital were evaluated if they had received at least one dose of pegaspargase and a vaccine against SARS‐CoV‐2. Data collected included pegaspargase doses and dates of administration, details of pegaspargase hypersensitivity reaction, anti‐pegaspargase antibody results, manufacturer of COVID‐19 vaccine administered, number and dates of vaccine doses, and vaccine reaction symptoms post‐vaccination. Clinical notes and allergy documentation were reviewed in the electronic health record for documentation of reaction to SARS‐CoV‐2 vaccine and history of hypersensitivity reaction to pegaspargase. This study was approved by the institutional review board.

The demographics of the 78 patients who had previously been treated with pegaspargase and received SARS‐CoV‐2 vaccine are listed in Table 1. Fifty‐two patients received at least one dose of anti‐SARS‐CoV‐2 vaccine at St. Jude and 26 patients received their vaccine elsewhere; documentation of administration for these patients was included in the electronic health record. One patient experienced a mild reaction to the Janssen vaccine approximately 25 minutes after administration that included abdominal pain, nausea, dizziness, sweating, and hypertension. These symptoms did not meet CDC criteria for an allergic reaction and resolved without interventions within 30 minutes. The other 77 patients had no adverse reactions to their SARS‐CoV2 vaccine.

**TABLE 1:** Patient demographics

Patient demographics	*N* = 78 (%)
Age at leukemia diagnosis (years)	
Median	9.1
Range	1.3–18.5
Age at time of first vaccine (years)	
Median	14.6
Range	5.2–25.8
Sex	
Male	47 (60.3)
Female	31 (39.7)
Self‐declared race/ethnicity	
White	51 (65.4)
Black	17 (21.8)
Hispanic	6 (7.7)
Asian	3 (3.8)
American Indian	1 (1.3)
Diagnosis	
B‐ALL	57 (73)
T‐ALL	15 (19.2)
Early T‐cell precursor‐ALL	2 (2.6)
B‐LLy	2 (2.6)
T‐LLy	2 (2.6)
Number of vaccine doses documented in St. Jude medical record	
1	22 (28.2)
2	46 (59)
3	10 (12.8)
Manufacturer of the vaccine	
Janssen	7 (9)
Moderna	6 (7.7)
Pfizer‐BioNTech	65 (83.3)
Time from last pegaspargase dose to first vaccine administration (Years)	
Median	3.7
Range	0.22–12.1
History of allergic reaction to pegaspargase, CTCAE version 3	9 (11.5%)
Grade 2	3 (33.3%)
Grade 3	6 (66.7%)
Pegaspargase antibodies positive at any time during treatment	24 (30.8%)
Time from last known positive antibodies to first vaccine dose (years)	
Median	4.3
Range	0.65–12.3
Pegaspargase antibodies remaining positive at last test performed	6 (7.6%)
Time from last known positive antibodies to first vaccine dose (years)	
Median	3.2
Range	1.5–7.7

Abbreviations: ALL, acute lymphoblastic leukemia; LLy, lymphoblastic lymphoma; CTCAE, common terminology criteria for adverse events.

Of the 24 patients with positive pegaspargase antibodies during their treatment for ALL, 9 experienced clinical hypersensitivity to pegaspargase. Five of these nine patients received an mRNA vaccine that contained PEG; none of them developed a reaction (Figure 1). Of the nine patients with hypersensitivity reaction to pegaspargase, three had tolerated subsequent pegaspargase infusion with desensitization (*n* = 2) or pre‐medication (*n* = 1). Six of twenty‐four patients, including four with hypersensitivity reactions, had persistently positive pegaspargase antibodies at last assessment 1.5 to 7.7 (median, 3.2) years before they received and tolerated their vaccine. Of these six patients, four with a history of hypersensitivity to pegaspargase received Janssen vaccine, and two without a history of hypersensitivity received Pfizer‐BioNTech vaccine.

**FIGURE 1:**
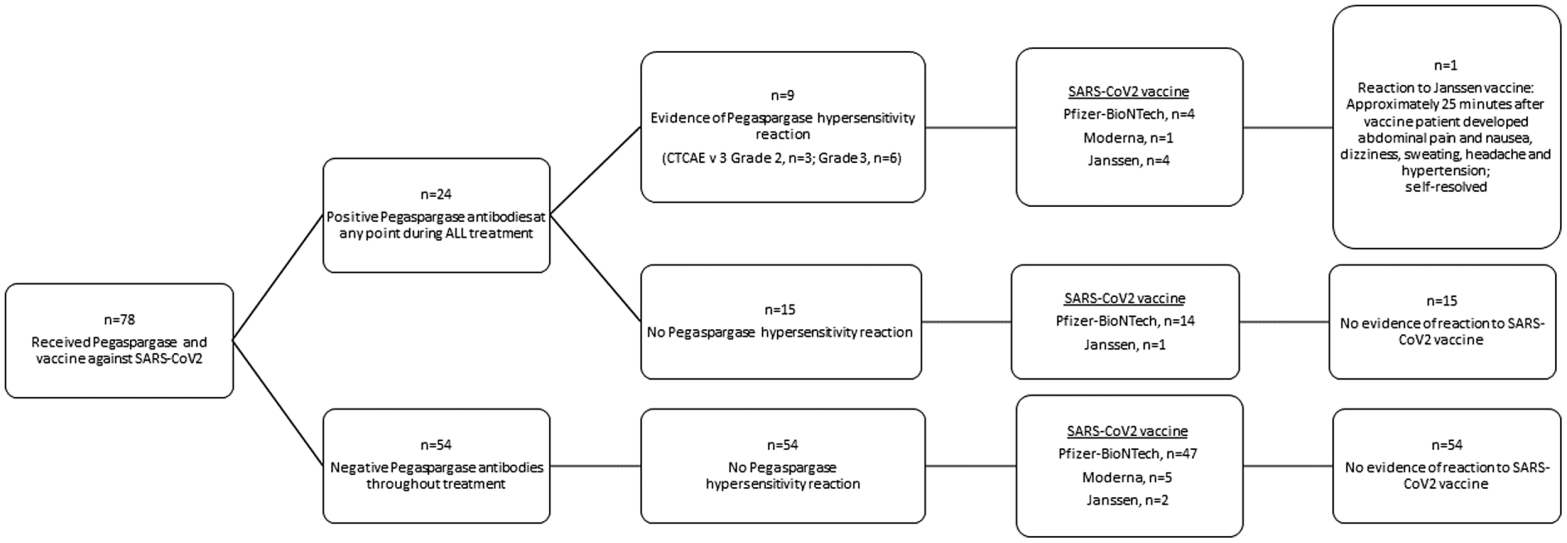
Vaccine administration based on the presence of pegaspargase antibodies and hypersensitivity reactions at any point during treatment.

Our data support the tolerability of PEG‐containing mRNA vaccines for SARS‐CoV‐2 in patients with a history of anti‐pegaspargase antibodies with or without a history of clinical hypersensitivity. Our finding is consistent with prior publications indicating mRNA vaccines for SARS‐CoV‐2 are safe to administer in patients with history of clinical pegaspargase allergic reactions.^12^, ^13^ Our data clarify that such administration is safe even in patients who had a prior immune reaction to PEG. PEG and polysorbate 80 have similar structure with polyether groups; cross reactivity of allergic reactions to PEG and polysorbate 80 have been reported.^15^, ^16^ Thus, cross reactivity to polysorbate in the vaccine manufactured by Janssen can occur in patients with prior pegaspargase hypersensitivity. It is possible such a cross‐reaction was the cause of the observed symptoms in one of our five patients with anti‐pegaspargase antibodies. These data support the recommendation that patients with hypersensitivity reaction to pegaspargase should also be considered for additional monitoring after Janssen vaccine administration. Our data also support the safety of vaccine administration in such patients.

Limitations of our study include a variable interval between anti‐pegaspargase antibody measurement and vaccination as well as a limited sample size, particularly for patients with a history of clinical allergic reaction. A strength of our data is the ability of available antibody data to confirm that patients had previously experienced true immune‐mediated hypersensitivity reactions to pegaspargase as opposed to infusion reactions, which may have similar clinical manifestations but are typically milder in degree.^7^ Taken together, our data indicate that neither a history of anti‐pegaspargase antibodies nor clinical hypersensitivity reaction should be a barrier for vaccination against SARS‐CoV‐2.

## AUTHOR CONTRIBUTIONS

HDS and SEK designed the study, analyzed data, performed analyses. All authors contributed data and participated in manuscript preparation and revision.

## CONFLICT OF INTEREST

SEK served as a consultant for Servier. The other authors indicate no relevant financial disclosures.

The data that support the findings of this study are available from the corresponding author upon reasonable request.

## Data Availability

The data that support the findings of this study are available from the corresponding author upon reasonable request.
